# Protein–protein interaction prediction based on ordinal regression and recurrent convolutional neural networks

**DOI:** 10.1186/s12859-021-04369-0

**Published:** 2021-10-08

**Authors:** Weixia Xu, Yangyun Gao, Yang Wang, Jihong Guan

**Affiliations:** 1 School of Information Management, Shanghai Lixin University of Accounting and Finance, No. 995 Shangchuan Road, Shanghai, 201209 China; 2 Shanghai Key Laboratory of Intelligent Information Processing, and School of Computer Science, Fudan University, No. 220 Handan Road, Shanghai, 200433 China; 3 Department of Computer Science and Technology, Tongji University, No. 4800 Caoan Road, Shanghai, 201804 China

**Keywords:** Protein–protein interaction, Confidence score, Ordinal regression, Recurrent convolutional neural network

## Abstract

**Background:**

Protein protein interactions (PPIs) are essential to most of the biological processes. The prediction of PPIs is beneficial to the understanding of protein functions and thus is helpful to pathological analysis, disease diagnosis and drug design etc. As the amount of protein data is growing fast in the post genomic era, high-throughput experimental methods are expensive and time-consuming for the prediction of PPIs. Thus, computational methods have attracted researcher’s attention in recent years. A large number of computational methods have been proposed based on different protein sequence encoders.

**Results:**

Notably, the confidence score of a protein sequence pair could be regarded as a kind of measurement to PPIs. The higher the confidence score for one protein pair is, the more likely the protein pair interacts. Thus in this paper, a deep learning framework, called ordinal regression and recurrent convolutional neural network (OR-RCNN) method, is introduced to predict PPIs from the perspective of confidence score. It mainly contains two parts: the encoder part of protein sequence pair and the prediction part of PPIs by confidence score. In the first part, two recurrent convolutional neural networks (RCNNs) with shared parameters are applied to construct two protein sequence embedding vectors, which can automatically extract robust local features and sequential information from the protein pairs. Based on it, the two embedding vectors are encoded into one novel embedding vector by element-wise multiplication. By taking the ordinal information behind confidence score into consideration, ordinal regression is used to construct multiple sub-classifiers in the second part. The results of multiple sub-classifiers are aggregated to obtain the final confidence score. Following that, the existence of PPIs is determined by the confidence score. We set a threshold $\theta$, and say the interaction exists between the protein pair if its confidence score is bigger than $\theta$.

**Conclusions:**

We applied our method to predict PPIs on data sets *S. cerevisiae* and *Homo sapiens*. Through experimental verification, our method outperforms state-of-the-art PPI prediction models.

## Background

Proteins [1, 2] are critical to the cells and tissues in the body. They participate in various life activities, like antibody immunity, catalyzing metabolic reactions and transporting molecules, etc. Usually, proteins are associated with other proteins to form the protein complexes, so as to perform the functions of living organisms in a better way. Among the protein complexes, protein-protein interactions (PPIs) play a crucial role in successfully carrying out different biological processes in cells, such as transcription, translation, cell cycle control, and secretion [3]. Therefore, the problem of PPI prediction is of great significance in pathological analysis [4], disease diagnosis [5], drug design [6], and is becoming a research focus in the field of proteomics.

A large amount of high-throughput experimental methods have been applied to predict the PPIs from protein complexes, such as Yeast double hybrid screens [7], tandem affinity purification [8, 9], and proteome chips and micro-array technology [10, 11]. However, with the accumulation of protein data, these methods suffer from the restrictions of time and economic cost, and cannot meet the needs of human life science research in the post genomic era. It is also worth noting that due to the existence of the subjective or objective factors, such as operation error and experimental error, the experimental results often deviate slightly from the actual results, sometimes even leading to a large proportion of false positive or false negative experimental data. For example, there are about 80,000 PPIs predicted by these high-throughput experimental methods, but only a relatively small number (about 2400) of these PPIs could be obtained by more than one method [7]. Hence, only using high-throughput experimental methods would not get high-quality and reliable experimental results. To overcome these drawbacks, computational methods have attracted researcher’s attention. They [12] predict the PPIs mainly based on the sequential information of amino acids of proteins. Besides, some other methods [13–15] are based on the structural information, or based on fusion of multiple information from different data sources. With these information, it is also necessary to extract effective features to guarantee good prediction results. For this purpose, researchers begin to focus on the protein sequence encoding techniques and the corresponding predicting techniques for PPIs.

Shen et al. [12] introduced a conjoint triad (CT) descriptor, which considered the properties of each amino acid and its neighbouring amino acids and extracted the features of the local environmental information in the amino acid sequence. Gough et al. [13] proposed a method to describe the amino acid sequence based on the physical and chemical properties, combined with the structural information of protein. Later based on the physical and chemical properties of amino acids, Guo et al. [14] established an auto covariance (AC) encoding method to get the correlation and the interaction information of amino acids at different positions. For the high dimension of features of protein sequences, Thanathamathee et al. [15] used a principal component analysis algorithm to reduce the dimension first, and then constructed a forward feedback neural network as a classifier to predict the PPIs. Recently, some deep learning frameworks were proposed. For instance, Hashemifar et al. [16] presented a Siamese-like convolutional neural network with random projection, and data augmentation technique was used to extract sequential information in the framework. Li et al. [17] discussed another deep neural network framework, which learned the local features automatically only from protein primary sequences, according to the encoding, embedding, convolutional neural network, and long short-term memory (LSTM) neural network layers.

Notably, confidence score could be regarded as a kind of measurement for PPIs. The higher confidence score one protein pair gets, the more likely the protein pair interacts. Thus in this work, we propose a novel method, called ordinal regression and recurrent convolutional neural network (OR-RCNN), to predict PPIs by confidence score. The method could be concluded into two parts: (1) an encoder of protein sequence pair based on recurrent convolutional neural network (RCNN), and (2) PPI prediction model based on ordinal regression. In order to deal with the protein sequence pair in a better way, two RCNNs with shared parameters are introduced here. Each RCNN encodes one of the protein sequence pair into an embedding vector, which integrates multiple convolution layers with pooling and bidirectional gate recurrent unit (GRU) layers with concatenate, so as to extract the local features and sequential information more accurately. An element-wise multiplication is then done on the two embedding vectors, encoding them into one novel embedding vector. Till now, the encoder of protein sequence pair is presented, which encodes a pair of protein sequences into one embedding vector aggregating multi-granularity features. In order to predict PPIs, we predict the confidence score of the embedding vector first. The ordinal information is hidden behind confidence score by means of artificially setting the ordinal sub-intervals of confidence score. To this end, the concept of rank is used to show the ordinal information, and each sub-interval is corresponding to a rank value. To efficiently use the ordinal information, ordinal regression is applied here. Based on it, the prediction problem of confidence score is transformed into a series of binary classification problems. Some multi-layer perceptrons are utilized in the binary classification to get the ordinal information about rank. Later, all the ordinal information are aggregated to get the final confidence score of the protein pair. Finally, whether the interaction between protein pair exists is determined by its confidence score. Experimental results show that our method could boost the performance of PPI prediction on both the *S. cerevisiae* and *Homo sapiens* data sets.

## Results

In this section, we first introduce two PPI data sets *S. cerevisiae* and *Homo sapiens*. Then, we give the experimental setting. Finally, the experimental results are presented.

### Data sets

The high throughput data sets *S. cerevisiae* and *Homo sapiens* are both from STRING database [18]. There is a confidence score for each pair of protein sequences in *S. cerevisiae* and *Homo sapiens*. Let $\overline{CS}_{min}=0$ and $\overline{CS}_{max}=1$ be the minimal and maximal values of confidence score for protein pairs, respectively. We separate the interval of confidence score (0, 1) into *K* sub-intervals with equal length. Let $K=20$, and the 20 sub-intervals are (0, 0.05), [0.05, 0.1),…, [0.95, 1). The protein pair is labeled *k*, if its confidence score belongs to the *k*-th sub-interval. Thus, both datasets are split into 20 subsets according to the sub-intervals.

We only consider the data with the length of protein sequence between 50 and 2000. For the data set *S. cerevisiae*, there are 1584 data in the sub-interval (0, 0.05), and 5360 data in the sub-interval [0.7, 0.75). Considering that there are too many data in the rest 18 sub-intervals, we randomly select 5400 data in each sub-interval. Thus, there are totally 104144 data actually used in the experiment. For the data set *Homo sapiens*, since the true data set is too big, we randomly select 5000 data in each sub-interval in our experiment. We randomly select 90% data in each sub-interval as the training data, and the left 10% data as the test data for both *S. cerevisiae* and *Homo sapiens*.

### Experimental settings

Since the protein sequences have different lengths from 50 to 2000, we extend the short protein sequence to a sequence with length 2000 by adding zero-padding technique [19]. Let the batch size be 768, and let $d=3$. The 3-max-pooling mechanism is applied in the pooling layer. The output of bidirectional GRU layer with concatenate operator is a 150-dimension vector.

The AMSGrad algorithm [20] is used to optimize the cross-entropy loss function *L* for each sub-classifier. In the algorithm, we set the learning rate to be 0.001, and set the exponential decay rates of $\beta _{1}$ and $\beta _{2}$ to be 0.9 and 0.999, respectively.

We first evaluate the performance of each sub-classifier to see if the ordinal information about label has been pick up correctly. The evaluation is based on different comparison types: comparison of all the sub-classifiers, comparison of pre-trained embedding methods, selection of key parameters, comparison of computing equation for confidence score and study on the impact of the ratio of training over test data. Five criterions are used in the evaluation, including accuracy, precision, sensitivity (recall), specificity and $F_{1}$ score. Then, we emphasize the fact that the concatenate operator in our method performs better than the residual shortcut operator. Finally, our method is compared with some existing methods to show its advantages, based on the mean absolute error (MAE), mean squared error (MSE) and the above five criterions.

### Experimental results

#### Comparison of sub-classifiers

**Fig. 1:**
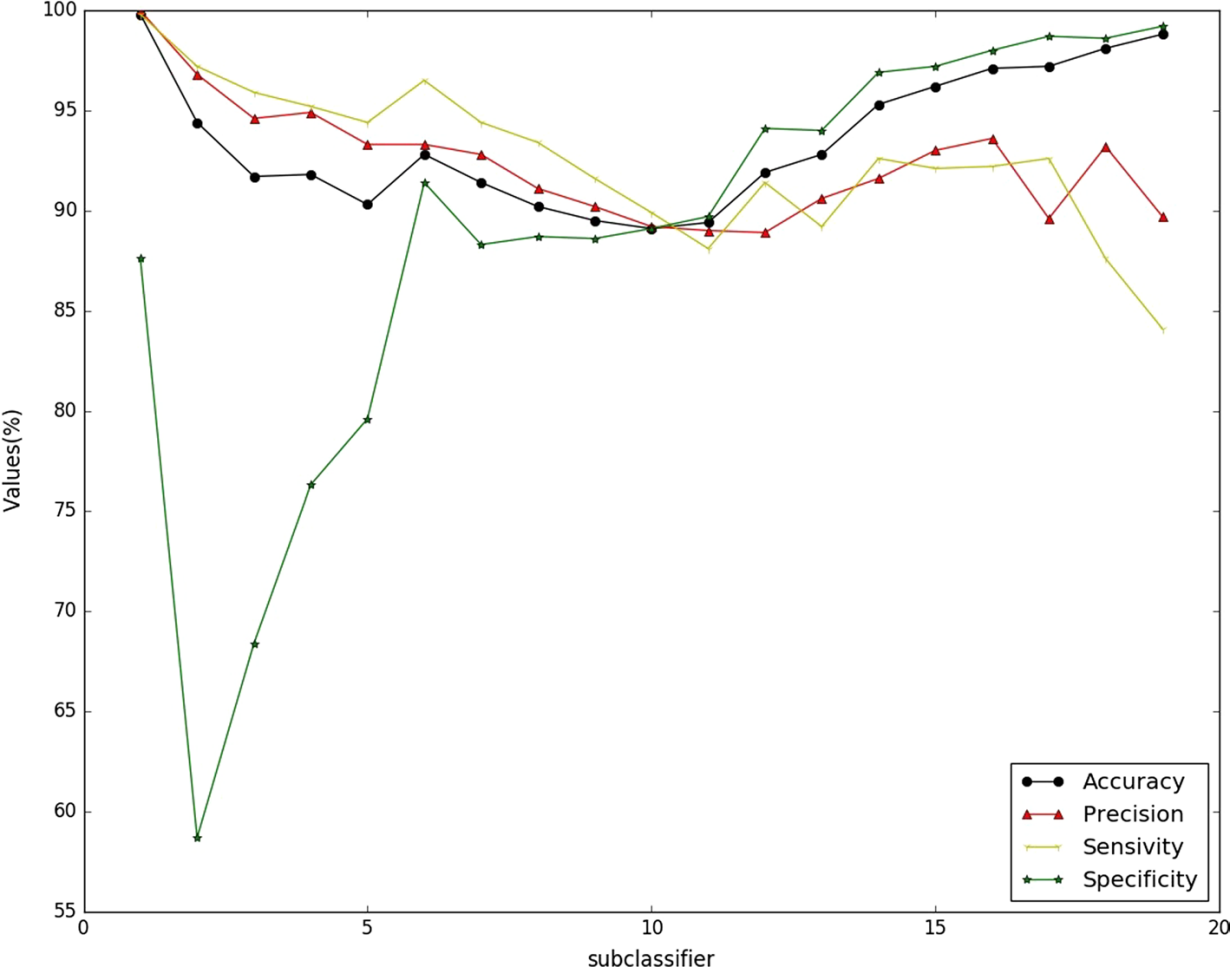
Performance of OR-RCNN for each sub-classifier

The performance of each sub-classifier for OR-RCNN method on data set *S. cerevisiae* is shown in Fig. 1. The 19 sub-classifiers have very close performance in accuracy, precision, sensitivity, and $F_{1}$ score. For the specificity, the first 5 sub-classifiers perform poorly while the last 14 sub-classifiers all have good performance. That is, when we get the ordinal information for lower labels, the sub-classifier is not a good choice for specificity, while for higher labels, it would be much better.

In the rest of the experiments, we will not show the performance for all the 19 sub-classifier, but show that for only three typical sub-classifiers $f_{5}, f_{10}, f_{15}$. The corresponding sub-classification problems are to predict if the label of a protein pair is bigger than 5, 10 and 15, respectively.

#### Comparison of pre-trained embedding methods

We mainly compare three embedding methods: (1) ${\mathbf{a}}_{co}$; (2) ${\mathbf{a}}_{eh}$; (3) one-hot. Let the half length *C* of context be 3, and let the size of negative sampling be 5. The embedding ${\mathbf{a}}_{co}$ is obtained by pre-training 8000 protein sequences of data set SHS148k from database STRING. The embedding ${\mathbf{a}}_{eh}$ is directly computed by the electrostaticity and hydrophobicity. The one-hot method assigns a 20 dimensional vector to each amino acid.

Table 1 shows the comparison results of performance of different embedding methods for sub-classifiers $f_{5}, f_{10}, f_{15}$ on data set *S. cerevisiae*, respectively. Obviously, the embedding methods ${\mathbf{a}}_{co}$ and ${\mathbf{a}}_{eh}$ get very close results to each other, while they outperform the one-hot method.

**Table 1:** Comparison of performance of pre-trained embedding methods for amino acids for sub-classifiers $f_{5}, f_{10}, f_{15}$

Embedding	Accuracy (%)	Precision (%)	Sensitivity (%)	Specificity (%)	$F_{1}$Score (%)
*Sub-classifier* $f_{5}$					
${\mathbf{a}}_{co}$	90.35	93.25	**94.42**	**79.61**	93.83
${\mathbf{a}}_{eh}$	**90.78**	**93.94**	94.21	79.60	**94.08**
One-hot	89.94	93.28	93.81	77.95	93.55
*Sub-classifier* $f_{10}$					
${\mathbf{a}}_{co}$	**89.15**	**89.24**	**89.90**	**89.05**	**89.57**
${\mathbf{a}}_{eh}$	88.86	88.85	89.77	88.88	89.31
One-hot	88.39	88.55	89.12	88.22	88.83
*Sub-classifier* $f_{15}$					
${\mathbf{a}}_{co}$	**96.17**	**93.03**	92.10	**97.25**	**92.57**
${\mathbf{a}}_{eh}$	95.51	90.63	**92.18**	**97.25**	91.40
One-hot	95.92	92.17	92.07	97.23	92.11

The values in each column represents the experimental results for each criterion of performance. Maximal values in each column is shown in bold

#### Selection of key parameters

There are two key parameters in the OR-RCNN method: (1) the dimension $d^{\prime}$ of hidden state, (2) the repeated times of RCNN unit. We study how to select the optimal parameters for our method.

Let the repeated times of RCNN unit be 5, we compare the prediction results for different dimensions of hidden state on data set *S. cerevisiae*. The result are shown in Table 2 for sub-classifiers $f_{5}, f_{10}, f_{15}$, respectively. We examine the performance with $d^{\prime}=10,25,50,75$ for each sub-classifier. As the dimension value increases from 10 to 50, the performance improves significantly. As it increases from 50 to 75, the performance improves only a little, or even decreased. Thus in most cases, $d^{\prime}=50$ is a better choice for our method.

**Table 2:** Comparison of performance with different dimensions of hidden states for sub-classifiers $f_{5}, f_{10}, f_{15}$

Dimension	Accuracy (%)	Precision (%)	Sensitivity (%)	Specificity (%)	$F_{1}$Score (%)
*Sub-classifier* $f_{5}$					
10	85.98	92.87	88.78	66.04	90.78
25	88.53	92.60	92.70	74.36	92.63
50	**90.35**	93.25	**94.42**	**79.61**	**93.83**
75	90.18	**94.21**	93.10	76.84	93.65
*Sub-classifier* $f_{10}$					
10	79.90	82.59	77.56	77.35	80.00
25	85.22	84.22	87.95	86.40	86.05
50	**89.15**	**89.24**	89.90	89.05	**89.57**
75	88.59	88.02	**90.27**	**89.24**	89.13
*Sub-classifier* $f_{15}$					
10	90.67	87.68	74.42	91.51	80.51
25	94.53	91.11	87.39	95.66	89.21
50	**96.17**	**93.03**	**92.10**	**97.25**	**92.57**
75	96.12	92.96	91.99	97.21	92.47

The values in each column represents the experimental results for each criterion of performance. Maximal values in each column is shown in bold

Given $d^{\prime}=50$, we investigate the influence of repeated times for RCNN unit on the OR-RCNN method. The repeated times is set from 1 to 5. Table 3 shows the compared results for sub-classifiers $f_{5}, f_{10}, f_{15}$ on data set *S. cerevisiae*, respectively. We can see that the more times the RCNN unit occurs in our method, the better performance it could achieve. However, when the repeated times range from 1 to 3, our method enhances the performance rapidly, and when repeated times range from 3 to 5, it enhances very slowly, or even reduce a little. Hence, we choose the best repeated times of RCNN unit to 5 in the experiments.

**Table 3:** Comparison of performance with different repeated times of RCNN unit for sub-classifiers $f_{5}, f_{10}, f_{15}$

Times	Accuracy (%)	Precision (%)	Sensitivity (%)	Specificity (%)	$F_{1}$Score (%)
*Sub-classifier* $f_{5}$					
1	82.31	88.55	88.72	60.39	88.64
2	88.19	92.88	91.85	72.59	92.25
3	89.29	93.01	93.23	76.16	93.12
4	90.00	**93.47**	93.69	77.77	93.58
5	**90.35**	93.25	**94.42**	**79.61**	**93.83**
*Sub-classifier* $f_{10}$					
1	78.62	77.10	83.56	80.57	80.20
2	84.86	86.09	84.41	83.58	85.24
3	87.71	88.05	88.25	87.34	88.15
4	88.29	88.27	89.27	88.32	88.77
5	**89.15**	**89.24**	**89.90**	**89.05**	**89.57**
*Sub-classifier* $f_{15}$					
1	81.38	69.28	50.50	84.20	58.42
2	92.30	81.32	91.21	96.79	85.98
3	95.86	91.84	**92.21**	**97.27**	92.03
4	95.90	92.70	91.36	97.00	92.03
5	**96.17**	**93.03**	92.10	97.25	**92.57**

The values in each column represents the experimental results for each criterion of performance. Maximal values in each column is shown in bold

#### Comparison of different computing equations for confidence score

The computing equation for confidence score is a critical step in our method. For any pair of protein sequences, the middle value of sub-interval where the predicted label falls in is taken to be the confidence score. Here, we compare this equation with other two, which take two endpoint values in the sub-interval, respectively,

1 $$CS_{1}(x_{i}) = 0.05 * (\bar{r}(x_{i}) -1),$$

2 $$CS_{2}(x_{i}) = 0.05 * \bar{r}(x_{i}),$$

where $\bar{r}(x_{i})$ is the predicted label for protein pair $x_{i}$. The compared results are presented in Table 4 on data set *S. cerevisiae*. Evidently, the MSE and MAE of our method are both lower than those of the Eqs. (1) and (2), implying that the selection of computing equation for confidence score would influence on the performance of OR-RCNN method. Moreover, the predicted confidence score for Eq. (6) approximates the true value more closely. Thus, we prefer Eq. (6) than the other two for our method.

**Table 4:** Comparison of different equations for confidence score

Eq.	MAE ($\times 10^{-2}$)	MSE ($\times 10^{-2}$)
*CS*	**6.131**	**1.155**
$CS_{1}$	6.640	1.161
$CS_{2}$	6.840	1.191

The values in each column represents the experimental results for each criterion of performance. Minimal values in each column is shown in bold

#### Study on the impact of the ratio of training set over test set

Given a data set, we have to split it into two subsets, one is the training set and the other is the test set. The ratio of training set over test set may influence the performance. Here, we check the impact of the ratio of training set over test set on performance with three sub-classifiers $f_{5}, f_{10}, f_{15}$, on *S. cerevisiae* data set. We set the ratio to 5:5, 6:4, 7:3, 8:2, 9:1, respectively, and the results are presented on Table 5. With the increasing of the ratio, the values of accuracy, precision, recall, specificity and F1-score all increase. Furthermore, the sub-classifier $f_{10}$ increases the fastest in the three sub-classifiers, $f_{15}$ increases a bit slower than $f_{10}$. Therefore, the ratio of training data and test data does impact on the performance of our method, and it achieves better performance with a bigger ratio. Besides, the ratio of training data over test data impacts differently on the three sub-classifiers.

**Table 5:** Study on the impact of the ratio of training over test data for sub-classifiers $f_{5}, f_{10}, f_{15}$

Proportion	Accuracy (%)	Precision (%)	Recall (%)	Specificity (%)	$F_{1}$Score (%)
*Sub-classifier* $f_{5}$					
5:5	88.54	92.20	93.14	75.16	92.67
6:4	88.71	92.33	93.21	75.48	92.77
7:3	89.23	92.75	93.44	76.49	93.10
8:2	89.78	93.11	93.80	77.76	93.45
9:1	**90.35**	**93.25**	**94.42**	**79.61**	**93.83**
*Sub-classifier* $f_{10}$					
5:5	82.88	83.92	82.80	81.78	83.36
6:4	84.63	84.75	85.75	84.49	85.25
7:3	85.83	85.71	87.16	85.95	86.43
8:2	86.95	86.88	88.09	87.02	87.48
9:1	**89.14**	**89.24**	**89.90**	**89.05**	**89.56**
*Sub-classifier* $f_{15}$					
5:5	93.32	87.94	85.99	95.14	86.95
6:4	93.83	88.60	87.41	95.62	88.00
7:3	94.63	90.63	88.44	95.99	89.52
8:2	95.05	91.57	89.10	96.22	90.32
9:1	**96.16**	**93.03**	**92.10**	**97.25**	**92.56**

#### Comparison of the operators concatenate and residual shortcut

In our method, the concatenate operator is applied to the bidirectional gated recurrent unit (GRU) layer. Here, we compare the performance of our method with the concatenate operator and that of the same method with residual shortcut operator. Table 6 exhibits the comparison results on Guo’s *Yeast* data set from database of interacting proteins (DIP) [21]. We can see that the accuracy, precision and F1-score of concatenate operator are all bigger than those of residual shortcut operator. Further, the accuracy of concatenate operator improves 1.6%, precision improves 1.25% and F1-score improves 1.74%, all compared with those of residual shortcut operator. It implies that the concatenate operator could improve the performance, thus it is more suitable for our method. In other words, the bidirectional GRU with concatenate enhances the delivering of features and makes use of the features much more efficiently.

**Table 6:** Comparison of the operators concatenate and residual shortcut on *Yeast* data set

Operator	Accuracy (%)	Precision (%)	F1 Score (%)
Concatenate	**97.23**	**97.29**	**97.24**
Residual Shortcut	95.63	96.04	95.50

The values in each column represents the experimental results for each criterion of performance. Maximal values in each column is shown in bold

#### Comparison with existing PPI prediction methods

In order to show the advantage of our method, we compare it with some state-of-art methods. Our method is an ensemble method and it consists of two modules: feature description model and prediction model. Here, we choose the methods AC [14] and composition transition distribution (CTD) descriptor [22] for feature description, and methods random forest (RF) [23], extreme gradient boosting (XGBoost) [24] and support vector machine (SVM) [25] for the prediction. Thus, we have the methods RF-AC, RF-CTD, XGBoost-AC, XGBoost-CTD, SVM-AC and SVM-CTD.

Table 7 demonstrates the comparison results of MAE and MSE for the confidence score on data sets *S. cerevisiae* and *Homo sapiens*. We can see that the RF-CTD method achieves the smallest MAE and MSE among the existing methods on both data sets, and the results of RF-AC is very close to those of RF-CTD. Meanwhile, our method reduces the MAE and MSE by 49.78% and 57.33% on data set *S. cerevisiae*, and reduces the MAE and MSE by 44.12% and 50.75% on data set *Homo sapiens*, respectively, both compared with RF-CTD. To sum up, we have following two conclusions: (1) Our method achieves much more accurate values of confidence score on both data sets, thereby improving the performance on different species; (2) The reductions of MAE and MSE vary with data sets, that is, our method improves the performance to different degrees on different species.

**Table 7:** Comparison with existing PPI prediction methods on different data sets

Data set	*S. cerevisiae*		*Homo sapiens*	
Methods	MAE($\times 10^{-2}$)	MSE($\times 10^{-2}$)	MAE($\times 10^{-2}$)	MSE($\times 10^{-2}$)
RF-AC	12.427	2.835	18.979	5.218
RF-CTD	12.210	2.707	18.850	5.163
XGBoost-AC	13.575	3.147	23.875	7.571
XGBoost-CTD	13.076	3.051	23.951	7.630
SVM-AC	24.466	8.025	–	–
SVM-CTD	31.174	14.435	–	–
OR-RCNN	**6.131**	**1.155**	**10.476**	**2.543**

The values in each column represents the experimental results for each criterion of performance. Minimal values in each column is shown in bold

**Fig. 2:**
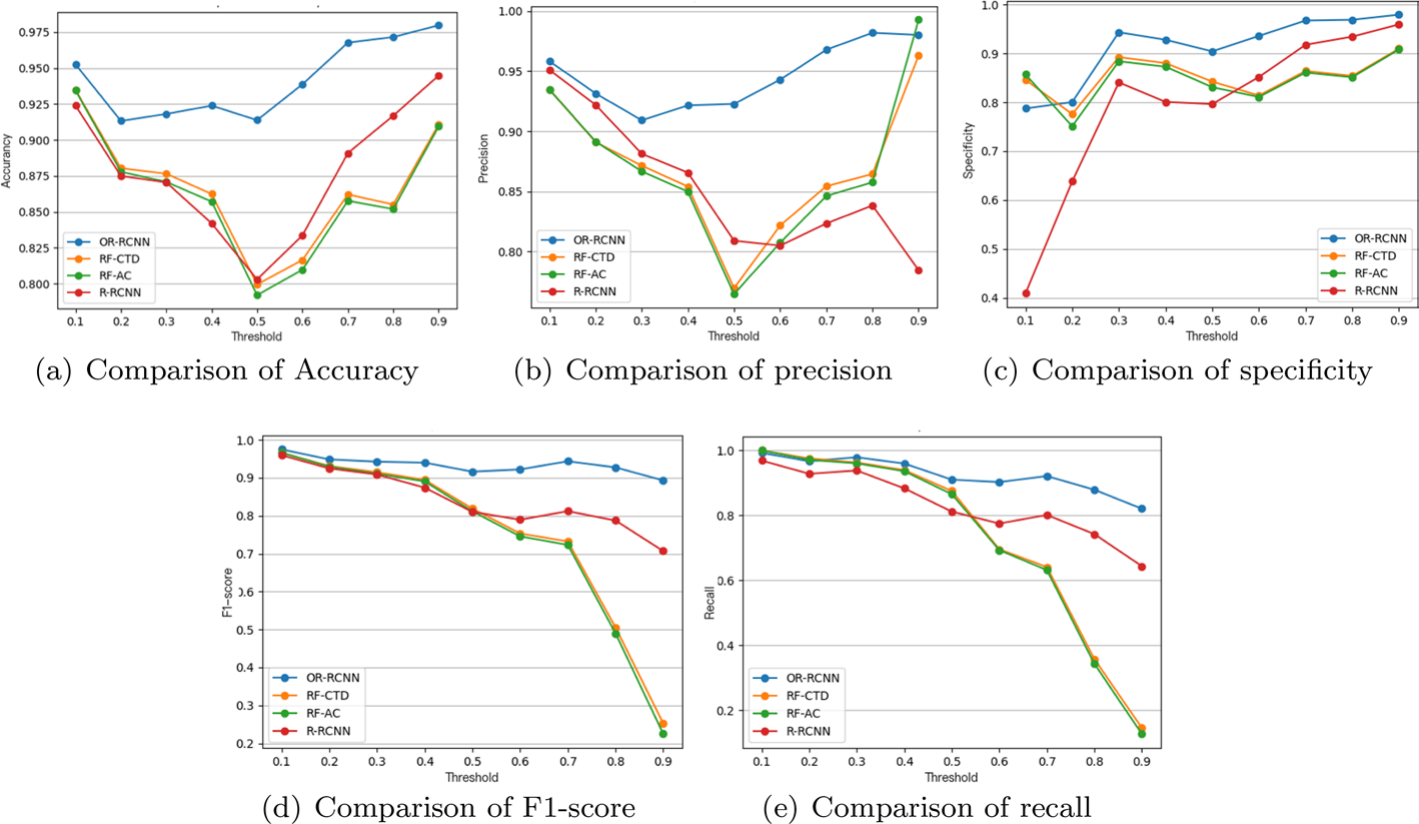
Comparison results when threshold $\theta$ is set to 0.1,0.2,…,0.9, respectively

After predicting the confidence score for the protein pairs, we set a threshold $\theta =0.1,0.2,\ldots ,0.9$, to predict the PPIs. Figure 2 illustrates the accuracy, precision, specificity, F1-score and recall of our method, compared with RF-AC, RF-CTD and R-RCNN methods. Note that R-RCNN is not an existing method. In order to show the effectiveness of ordinary regression in our method, the R-RCNN method is introduced. It consists of two parts. The first part is the RCNN encoder, which is the same as that of our method. While the second part R represents the regression model. Here, we use the multi-layer perceptron with scalar output as the regression model. In most cases, our method could get better results for the five criterions than other methods. Concretely, when the value of $\theta$ increases from 0.1 to 0.9, on one hand, the values of accuracy, precision and specificity decrease first and then increase, while those of F1-score and recall tend to decrease slowly. On the other hand, the values of the five criterion of our method fluctuate in a smaller range compared with the other three methods. Especially when we choose the value of $\theta$ ranging from 0.5 to 0.9, our method improves the performance more significantly. In general, our method outperforms most of the existing methods.

## Discussion

It is well known that most of the PPI prediction models contain two modules: one is an encoder encoding the protein pairs into feature vectors, the other is a prediction model determining whether the interactions exist in the protein pairs. Inspired by the idea of RCNN encoder and ordinal regression, we propose the OR-RCNN method to predict PPIs. On one hand, two RCNN encoders with shared parameters are assembled to one encoder, so that each protein pair could be encoded into one feature vector. For the encoder, we also substitute the concatenate operator for the residual shortcut operator in the bidirectional GRU layer, since experimental results on Guo’s Yeast data set have shown the advantages of concatenate operator. On the other hand, considering the fact that the higher the confidence score of one protein pair is, the more likely the protein pair interacts, we suggest to mine the hidden ordinal information behind the confidence score to boost the performance of PPI prediction. For this purpose, the ordinal regression is applied in our method. Compared to the common regression model without using ordinal information, the ordinal regression model improves the performance in terms of five metrics: accuracy, precision, specificity, F1-score and recall. In summary, by combining the assembled RCNN encoder and the ordinal regression model, our OR-RCNN method significantly boosts the prediction performance, and outperforms most of the existing methods.

## Conclusion

In this paper, an OR-RCNN method is proposed to predict PPIs according to its confidence score. In our method, the protein sequence pair is first encoded into one embedding vector based on two RCNN encoders. They share the same parameters, so as to reduce the complexity for training process. Next, multiple sub-classifiers are investigated to the embedding vector based on the idea of ordinal regression. It effectively exploits the ordinal information behind the confidence score by uniformly splitting the confidence interval into several non-overlapping sub-intervals, and rearranging the sub-intervals in an increasing order. Then, the ordinal information from these sub-classifiers of any protein pair are aggregated to get its confidence score. Finally, we predict the PPI of the protein pair with a threshold. Experiments have shown that the OR-RCNN method outperforms the state-of-the-art methods on data sets *S. cerevisiae* and *Homo sapiens*.

## Methods

In this section, we describe the OR-RCNN method for PPI prediction task. Some basic concepts of the RCNN encoder are introduced first. Then, the general framework of our method is conducted. Finally, the technical details of our method are presented.

### Preliminaries

Denote by ${\mathcal{A}}$ the vocabulary of 20 standard amino acids. Denote by $S=[a_{1}, \ldots , a_{l}]$ the sequence of amino acids for a protein, where $a_{i}$ is an amino acid in the vocabulary.

#### Pre-trained embedding for amino acids

Since the sequential information of amino acids for a protein is usually non-numerical, the embedding method is necessary in the pre-training process. An amino acid $a \in {\mathcal{A}}$ could be embedded into a semi-latent vector ${\mathbf{a}}$, and ${\mathbf{a}}$ is numerical.

Here, we introduce two embedding methods. The first method applies the Skip-Gram model [26] to the protein sequence. Let ${\mathbf{a}}_{co}$ be the embedding, which measures the similarity of co-occurrence of two amino acids. Formally, to maximize the average log probability of the similarity, we minimize the objective function $J_{SG}$

$$\begin{aligned} J_{SG} = - \frac{1}{|S|} \sum _{a_{t} \in S} \sum _{-C \le j \le C, j\ne 0} \log p({\mathbf{a}}_{co,t+j} | {\mathbf{a}}_{co,t}), \end{aligned}$$

where ${\mathbf{a}}_{co,t}$ and ${\mathbf{a}}_{co,t+j}$ are both the embedding results for the *t*’th amino acid $a_{t} \in S$ and the neighbor, respectively, and *C* is the length of the half context. Note, the context is a subsequence of the protein sequence *S* with length $2C+1$. The probability *p* is a softmax function:

$$\begin{aligned} p({\mathbf{a}}_{co,t+j} | {\mathbf{a}}_{co,t}) = \frac{\exp ({\mathbf{a}}_{co,t+j} \cdot {\mathbf{a}}_{co,t})}{\sum _{k=1}^{m}\exp ({\mathbf{a}}^{\prime}_{co,k} \cdot {\mathbf{a}}_{co,t})}, \end{aligned}$$

where ${\mathbf{a}}_{co,k}^{\prime}$ is a negative sample not occurring in the same context with ${\mathbf{a}}_{co,t}$, and *m* is the size of negative sampling.

The second method [12] expresses the embedding as ${\mathbf{a}}_{eh}$. It measure the similarity of properties, like electrostaticity and hydrophobicity, between two amino acids. The reason is that electrostatic and hydrophobic interactions occupy the most important position in PPIs. They could be computed by their dipoles and volumes of the side chains of amino acids, respectively. Naturally, the 20 amino acids in ${\mathcal{A}}$ are divided into 7 classes. Thus, ${\mathbf{a}}_{eh}$ is a 7 dimensional vector, like one-hot encoding method.

#### RCNN encoder

**Fig. 3:**
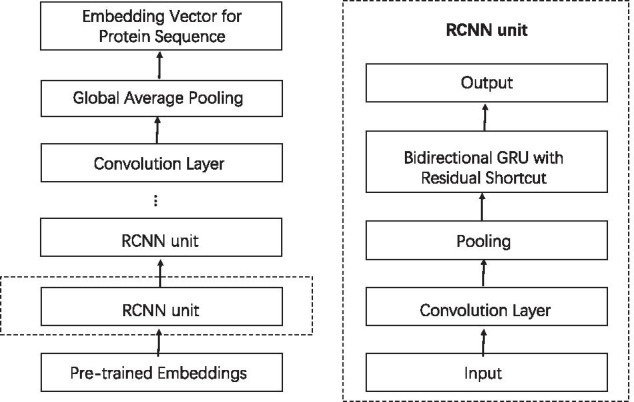
Illustration of RCNN encoder. The left illustrates the whole structure of RCNN encoder, and the right exhibits the structure of RCNN unit

RCNN encoder [27] is applied to get the global sequential information and local features which are both crucial to predict PPIs. In the deep neural network encoder framework, there are mainly two computing modules. One is the convolution layer with pooling, and the other is the bidirectional GRU with residual. The general framework is illustrated in Fig. 3 [27].

*The convolution layer with pooling* The purpose of the convolution layer with pooling is to extract local information from the input. Let $S^{\prime} = [v_{1}, v_{2}, \ldots , v_{l}]$ be an input sequence of pre-trained embedding for a protein or the output of a previous neural network layer. We sample a consecutive sub-sequence $[v_{t}, v_{t+1}, \ldots , v_{t+d-1}]$ (simply denoted by $v_{t:t+d-1}$) from $S^{\prime}$. By using the weight-sharing kernel $M \in {\mathbb{R}}^{d \times d^{\prime}}$, it generates a $d^{\prime}$ dimensional latent vector $h_{t}^{1}$

$$\begin{aligned} h_{t}^{1} = Conv (v_{t:t+d-1}) = M v_{t:t+d-1} + b_{M}, \end{aligned}$$

from the sub-sequence $v_{t:t+d-1}$, where *d* is the parameter for the kernel size and $b_{M}$ is a vector for bias. The latent vector $h_{t}^{1}$ extracts local features from the sub-sequence $v_{t:t+d-1}$. Let $t=1,2,\ldots ,l-d+1$, respectively, it obtains a sequence of latent vectors $H = [h_{1}^{1}, h_{2}^{1}, \ldots , h_{l-d+1}^{1}]$, generating from all the sub-sequences of input sequence $S^{\prime}$. And *H* is the output of convolution layer.

Consider that the size of *H* is too big, i.e., there are too many features extracted from the input. Thus, in the pooling layer, it aims at reducing the dimension of the output *H* to make it robust. To this end, the “*n*-max-pooling mechanism” [28, 29] is employed to every sub-sequence sampled from *H* with length *n*, where the length *n* is a pre-defined parameter. Notably, any two sub-sequences sampled from *H* are not overlapped. The mechanism is to choose the maximal value of the subsequence as its value in each dimension *j*, defined as

$$\begin{aligned} h_{t,j}^{2} = \max (h_{t,j}^{1}, h_{t+1,j}^{1}, \ldots , h_{t+n-1,j}^{1}), \end{aligned}$$

Though the pooling layer discretizes the output of convolution layer, the most important features to the subsequence are preserved in the pooling output, and the number of preserved features is only 1/*n* of that of output of convolution layer.

*Bidirectional GRU with Residual* The GRU [30, 31] is an alternative of the long short-term memory (LSTM) network. Compared with LSTM, the GRU is much more efficient, and it discovers the sequential information without the demand of single memory cells [32]. For the purpose, each unit is composed of two kinds of gates: one is the reset gate $r_{t}$, and the other is the update gate $z_{t}$.

Given an input vector $v_{t} \in S^{\prime}$, GRU updates the hidden state $h_{t}^{3}$ based on the weighted average value of the candidate state $\tilde{h}_{t}^{3}$ and the previous state $h_{t-1}^{3}$. The updating equation is expressed as follows

$$\begin{aligned} h_{t}^{3} &= GRU(v_{t}) = z_{t} \odot \tilde{h}_{t}^{3} + (1-z_{t}) \odot h_{t-1}^{3}, \\ z_{t} &= \sigma (M_{z}v_{t} + N_{z}h_{t-1}^{3} + b_{z} ), \\ \tilde{h}_{t}^{3} &= \tanh (M_{s}v_{t} + r_{t} \odot (N_{s}h_{t-1}^{3}) + b_{s} ), \\ r_{t} &= \sigma (M_{r}v_{t} + N_{r}h_{t-1}^{3} + b_{r} ), \end{aligned}$$

where $M_{*}$ and $N_{*}$ ($* \in \{z,s,r\}$) are weight matrices, $b_{*}$ is a bias vector, $\sigma$ is a sigmoid function, and the notation $\odot$ means the element-wise multiplication. Here, the reset gate $r_{t}$ calculates the candidate state $\tilde{h}_{t}^{3}$, and the update gate $z_{t}$ updates the hidden state $h_{t}^{3}$.

The bidirectional GRU layer [27] takes into account the sequential information of the input sequence $S^{\prime}$ in two directions. In the forward encoding process $\overrightarrow{GRU}$, the input sequence $[v_{1}, v_{2}, \ldots , v_{l}]$ is read from $v_{1}$ to $v_{l}$. While in the backward encoding process $\overleftarrow{GRU}$, it is read from $v_{l}$ to $v_{1}$. For every input vector $v_{t}$, the two encoding results for different directions are put together, that is,

3 $$\begin{aligned} h_{t}^{4} = BiGRU(v_{t}) = [\overrightarrow{GRU}(v_{t}), \overleftarrow{GRU}(v_{t})]. \end{aligned}$$

In addition, the residual mechanism [33] is also implemented in the bidirectional GRU layer. It identically maps the bidirectional GRU input to its output with a residual shortcut. Thus, the value of input vector $v_{t}$ is added to the hidden state $h_{t}^{4}$, and the bidirectional GRU layer with residual shortcut is defined as

4 $$\begin{aligned} h_{t}^{5} = ResGRU(v_{t}) = [\overrightarrow{GRU}(v_{t})+v_{t}, \overleftarrow{GRU}(v_{t})+v_{t}]. \end{aligned}$$

It greatly simplifies the training process, and requires much less time for updating parameters to converge.

*Protein Sequence Encoding* Figure 3(left) shows the general framework of the encoding process for a given protein sequence *S*, denoted by $E_{RCNN}(S)$.

Given a protein sequence *S*, the convolution layer with pooling and the bidirectional GRU layer with residual shortcut occur in the framework alternately. The convolution layer is the first encoding layer to extract the local features from the input sequence, and the pooling layer is to make the convolution result robust. Then, the robust results are input into the bidirectional GRU layer with residual, such that the sequential information are preserved. The two components form a RCNN unit, illustrated in Fig. 3(right). By using multiple RCNN units, we can get a multi-granular feature aggregation for the protein sequence *S*. Indeed, before the RCNN unit, the protein sequence *S* has been embedded into one vector, and it is the feature vector of the protein. By virtue of the first RCNN unit, the feature vector is encoded into another vector by Eq. (4), that is, the features are aggregated for the first time. Then, the aggregated vector is regarded as the input of the second RCNN unit, and it is aggregated again. In other words, the features of protein sequence S are aggregated as many times as the repeating occurrence of the RCNN unit.

On top of the framework, the last bidirectional GRU layer is followed by a convolution layer with pooling. The convolution layer is the same to that in RCNN unit, such that the local features are extracted from the final hidden states $H^{\prime} = [h_{1}^{\prime}, h_{2}^{\prime}, \ldots , h_{|H^{\prime}|}^{\prime} ]$. However, the pooling layer differs from that in RCNN unit. Instead of the “*n*-max-pooling mechanism”, the “global average pooling mechanism” [34] is applied here, since the dimensions of the final hidden states and the previous hidden states are not necessary to be equal. It takes the average of all the features, i.e.,

$$\begin{aligned} E_{RCNN}(S) = \frac{1}{|H^{\prime}|} \sum _{t=1}^{|H^{\prime}|} h_{t}^{\prime}. \end{aligned}$$

This is the result of protein sequence encoder.

### Overview

**Fig. 4:**
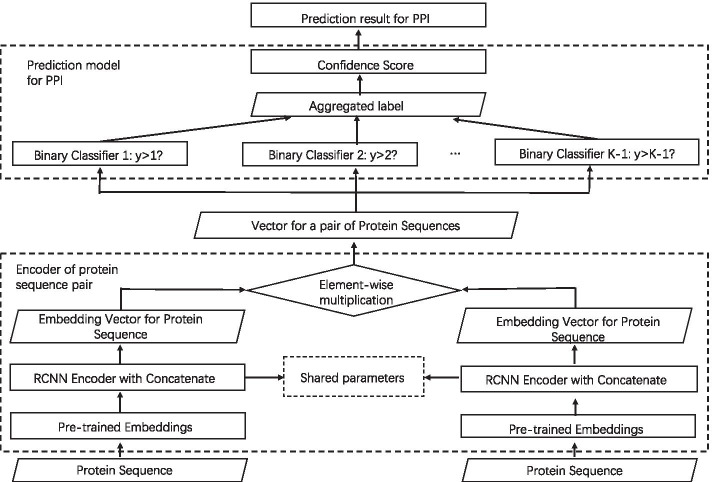
Illustration of the general framework for OR-RCNN method

The framework of OR-RCNN method is illustrated in Fig. 4. It is composed of two parts: one is the encoder for protein sequence pair (in the bottom dashed rectangle), the other is the prediction model for PPIs by confidence score.

The encoder for protein sequence pair contains two RCNNs with shared parameters and the element-wise multiplication technique. Each RCNN encodes one sequence of the protein pair into an embedding vector. Since the two RCNNs are both deep neural networks, they share the same parameters to reduce the computational complexity of the training process. Based on it, we use the element-wise multiplication technique to transform the two embedding vectors into one vector. In other words, the protein sequence pair is encoded into one embedding vector.

In order to predict PPI for the protein sequence pair, we use the confidence score to measure the likelihood of existence of PPI. The higher confidence score the protein sequence pair has, the more likely the protein pair interacts. Thus, the problem of PPI prediction is converted to the problem of the confidence score prediction. Given ${\mathcal{N}}$ protein pairs and the corresponding confidence scores, we first divide the interval of confidence score value into *K* sub-intervals. Obviously, the sub-intervals could be ranked in an increasing order. After ranking, we give the *k*’th ($k=1,2,\ldots , K$) sub-interval a label *k*. Thus, each protein pair is labeled *k* ($k=1,2,\ldots , K$), if its value of confidence score falls in the *k*’th sub-interval. To exploiting the ordinal information in a better way, the ordinal regression is investigated here. It trains $K-1$ binary sub-classifiers by the ${\mathcal{N}}$ protein pairs and their labels. When a novel protein pair is coming, the $K-1$ binary sub-classifiers could be jointly used to predict the final label for the protein pair. Based on it, the label of the protein pair is mapped into the value of confidence score by a certain computing equation. Finally, the PPI prediction result is totally determined by the confidence score.

### Technical details

In order to simplify the notations, let $x_{i} = (S_{i_{1}}, S_{i_{2}}), i=1,\ldots ,{\mathcal{N}}$ be ${\mathcal{N}}$ pairs of proteins, and $\overline{CS}_{i}$ be the confidence score of $x_{i}$.

#### Encoder for a pair of protein sequences

Given a pair of protein sequences $x_{i} = (S_{i_{1}}, S_{i_{2}})$, the protein sequences $S_{i_{1}}$ and $S_{i_{2}}$ are first embedded into a vector, respectively, in the pre-training process. And the embedding vector is the feature vector of the corresponding protein. This step ensures the input proteins $S_{i_{1}}$ and $S_{i_{2}}$ are both numerical type, preparing for the follow-up work.

**Fig. 5:**
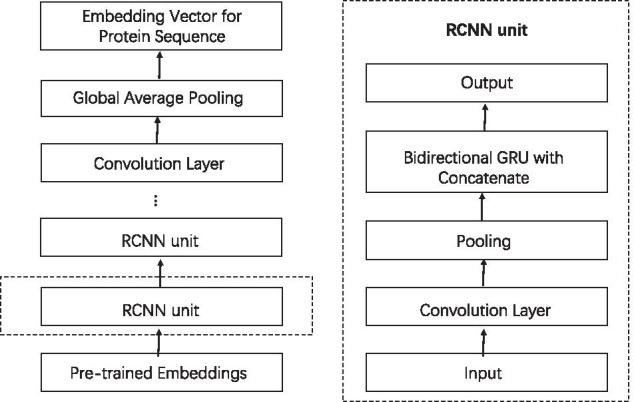
Illustration of RCNN encoder with concatenate operator. The left is the structure of RCNN encoder with concatenate operator, and the right is the structure of RCNN unit

Then, the two embedding vectors are both encoded to another vectors, respectively, by two RCNNs with concatenate operator. Note that, the RCNN with concatenate operator is a slightly modification of RCNN encoder with residual shortcut (Eq. (4)) [27]. Figure 5 shows the workflow of RCNN encoder with concatenate operator.

Our method only differs in the RCNN unit for the bidirectional GRU layer. Our method use the concatenate operator [35], instead of the residual mechanism. It connects all the features on the channels to realize the feature reuse. Given an input vector $v_{t}$ of the convolution layer and the hidden state $h_{t}^{4}$ (Eq. (3)), the concatenate operator is defined as follows

5 $$\begin{aligned} h_{t}^{5} = ConcatGRU(v_{t}) = [\overrightarrow{GRU}(v_{t}), \overleftarrow{GRU}(v_{t}), v_{t}]. \end{aligned}$$

Here, the input vector $v_{t}$ is concatenated to the right side of hidden state $h_{t}^{4}$, so as to avoid the problem of gradient disappearing. Furthermore, it enhances the delivering of features, makes use of the features much more efficiently, and reduces the numbers of parameters to a certain extent. In other words, the protein sequences $S_{i_{1}}$ and $S_{i_{2}}$ are encoded to the $E_{RCNN}(S_{i_{1}})$ and $E_{RCNN}(S_{i_{2}})$, respectively, by virtue of Eq. (5) in the bidirectional GRU layer. It is noteworthy that the two protein sequence encoders share the same parameters, which also reduces the number of parameters in our method, thereby reduces the computational cost.

Finally, the embedding vectors for protein sequences $E_{RCNN}(S_{i_{1}})$ and $E_{RCNN}(S_{i_{2}})$ are transformed into one vector by the element-wise multiplication, i.e., $E_{RCNN}(S_{i_{1}}) \odot E_{RCNN}(S_{i_{2}})$ (or simply write as $\bar{x}_{i}$). This multiplication is a common technique to discover the relationship between the two embedding vectors.

#### Prediction model for PPIs by confidence score

Suppose the value of confidence score for a protein pair falls in the interval $(\overline{CS}_{min}, \overline{CS}_{max})$. We separate it uniformly into *K* non-overlapped sub-intervals. The first sub-interval is expressed as $(\overline{CS}_{min}, \overline{CS}_{min}+(\overline{CS}_{max}-\overline{CS}_{min})/K)$, and the *k*’th ($k=2,\ldots ,K$) sub-interval is $[\overline{CS}_{min}+(k-1)(\overline{CS}_{max}-\overline{CS}_{min})/K, \overline{CS}_{min}+k(\overline{CS}_{max}-\overline{CS}_{min})/K)$. Accordingly, the *K* sub-intervals are ranked in an increasing order. Then, the label $y_{i}$ of $x_{i}$ is set to be *k* ($k=1,2,\ldots ,K$) automatically, if the confidence score of $x_{i}$ falls in the *k*’th sub-interval. Above all, $D =\{(\bar{x}_{i},y_{i}), i=1,\ldots , {\mathcal{N}} \}$ is the training data set in the prediction model for PPIs by confidence score.

Now, we begin to train the prediction model with data set *D*. Since the ordinal information of each data $\bar{x}_{i}$ is hidden behind the label $y_{i}$, ordinal regression [36, 37] is applied here to make full use of the ordinal information. The ordinal regression could be regarded as the aggregation of $K-1$ sub-classification problem, where the *k*’th ($k=1,2,\ldots ,K-1$) sub-classification problem is represented as determining whether the label of $x_{i}$ is bigger than *k*. To this end, we divide the whole training set *D* into two subsets: the positive class $D_{k}^{+}$ with the label bigger than *k*, and the negative class $D_{k}^{-}$ with the label no more than *k*, and then relabel them by

$$\begin{aligned}&D_{k}^{+} = \{ (\bar{x}_{i}, +1) | y_{i} > k \}, \\&D_{k}^{-} = \{ (\bar{x}_{i}, -1) | y_{i} \le k \}. \end{aligned}$$

Denote by $f_{k}$ ($k=1,2,\ldots ,K-1$) the sub-classifier for the *k*’th sub-classification problem. Obviously, the $K-1$ binary sub-classifiers $f_{k}, k = 1, 2, \ldots , K-1$ are all trained on the entire training set *D* with different divisions. It would contribute to getting better classification performance and can effectively avoid the over-fitting.

While training, the sub-classifier $f_{k}$ is determined by a multi-layer perceptron with a Leaky ReLU active function [38]. It solves the problem of gradient dispersion, and converges much faster than sigmoid or tanh active functions. Given a pair of protein sequences $x_{i}$, the output of the perceptron is a two dimensional vector, denoted by $\hat{s}^{i} =(\hat{s}_{1}^{i},\hat{s}_{2}^{i})$, and is normalized to another vector by softmax function, denoted by $s^{i}=(s_{1}^{i},s_{2}^{i})$,

$$\begin{aligned} s_{j}^{i} = \frac{\exp (\hat{s}_{j}^{i})}{\exp (\hat{s}_{1}^{i}) + \exp (\hat{s}_{2}^{i})}, j =1,2. \end{aligned}$$

Here, $s_{1}^{i}$ and $s_{2}^{i}$ represent the confidence level of $x_{i}$ belonging to the positive and negative classes, respectively. For the perceptron, the learning target is to minimize the cross-entropy loss function *L*,

$$\begin{aligned} L = -\frac{1}{|D|} \sum _{(\bar{x}_{i}, y_{i}) \in D} (q_{1}^{i} \log s_{1}^{i} + q_{2}^{i} \log s_{2}^{i}) \end{aligned}$$

where $q^{i} =(q_{1}^{i}, q_{2}^{i})$ is an one-hot indicator for the class label of $\bar{x}_{i}$. Then, we have the sub-classifier $f_{k}$

$$\begin{aligned} f_{k}(\bar{x}_{i}) = \left\{ \begin{aligned} 1,\quad s_{1}^{i} > s_{2}^{i} ,\\-1,\quad \mathrm{otherwise}. \end{aligned}\right. \end{aligned}$$

If the inequality $f_{k}(\cdot ) > 0$ holds true, it means the predicted label of the protein pair is bigger than *k*, otherwise, it is no more than *k*.

Now, we summarize all the ordinal information from each sub-classifier $f_{k}, k = 1,2,\ldots , K-1$ to derive the order of a given protein pair $x_{i}$. The $K-1$ outputs $f_{k}(\bar{x}_{i}), k = 1,2,\ldots , K-1$ are aggregated to predict the final label of $x_{i}$. The final label is defined as

$$\begin{aligned} \bar{r}(x_{i}) = 1 + \sum _{k=1}^{K-1} [f_{k}(\bar{x}_{i}) > 0], \end{aligned}$$

where $[\cdot ]$ is equal to 1, if the condition in $[\cdot ]$ is satisfied, otherwise it is equal to 0. Moreover, the predicted confidence score for protein pair $x_{i}$ is expressed as

6 $$\begin{aligned} CS(x_{i}) = \overline{CS}_{min} + (\overline{CS}_{max} -\overline{CS}_{min})/K * (\bar{r}(x_{i}) -1) +(\overline{CS}_{max}-\overline{CS}_{min})/(2K), \end{aligned}$$

which takes the middle value of the sub-interval corresponding to the predicted final label as its confidence score.

Finally, we predict PPI of protein pair $x_{i}$. Given a threshold $\theta$, if the predicted confidence score of $x_{i}$ is bigger than $\theta$, we determine there exists an interaction between the protein pair $x_{i}$, otherwise, there does not exist the interaction. Note that, the final prediction result is up to the value of threshold $\theta$. We could adjust the prediction performance by setting the optimal value of $\theta$ through experiments.

## Data Availability

The data sets used and/or analysed in this study are available from the corresponding articles. Three data sets *S. cerevisiae*, *Homo sapiens* and Yeast are all available at https://github.com/xuweixia88/OR-RCNN.git.

## Abbreviations

PPI: Protein protein interaction;
OR-RCNN: ordinal regression and recurrent convolutional neural network;
RCNN: recurrent convolutional neural network;
CT: conjoint triad;
AC: auto covariance;
GRU: gate recurrent unit;
MAE: mean absolute error;
MSE: mean squared error;
DIP: database of interacting proteins;
CTD: composition transition distribution;
RF: random forest;
XGBoost: extreme gradient boosting;
SVM: support vector machine;
LSTM: long short-term memory.
